# Harmonisation of PET/CT contrast recovery performance for brain studies

**DOI:** 10.1007/s00259-021-05201-w

**Published:** 2021-01-31

**Authors:** E. E. Verwer, S. S. V. Golla, A. Kaalep, M. Lubberink, F. H. P. van Velden, V. Bettinardi, M. Yaqub, T. Sera, S. Rijnsdorp, A. A. Lammertsma, R. Boellaard

**Affiliations:** 1 Department of Radiology & Nuclear Medicine, Amsterdam University Medical Centers, location VUmc, De Boelelaan 1117, 1081 HV Amsterdam, The Netherlands; 2 Department of Medical Technology, North Estonia Medical Centre Foundation, Tallinn, Estonia; 3 EANM Research Limited (EARL), Vienna, Austria; 4 Department of Surgical Sciences / Nuclear Medicine & PET, Uppsala University, Uppsala, Sweden; 5 Department of Radiology, Leiden University Medical Center, Leiden, The Netherlands; 6 IRCCS Scientific Institute San Raffaele Hospital, Milan, Italy; 7 Department of Nuclear Medicine, University of Szeged, Szeged, Hungary; 8 Department of Medical Physics, Catharina Hospital Eindhoven, Eindhoven, The Netherlands

**Keywords:** Brain, Neuroimaging, PET, Harmonisation, Standardisation, Image quality

## Abstract

**Purpose:**

In order to achieve comparability of image quality, harmonisation of PET system performance is imperative. In this study, prototype harmonisation criteria for PET brain studies were developed.

**Methods:**

Twelve clinical PET/CT systems (4 GE, 4 Philips, 4 Siemens, including SiPM-based “digital” systems) were used to acquire 30-min PET scans of a Hoffman 3D Brain phantom filled with ~ 33 kBq·mL^−1^ [^18^F]FDG. Scan data were reconstructed using various reconstruction settings. The images were rigidly coregistered to a template (voxel size 1.17 × 1.17 × 2.00 mm^3^) onto which several volumes of interest (VOIs) were defined. Recovery coefficients (RC) and grey matter to white matter ratios (GMWMr) were derived for eroded (denoted in the text by subscript e) and non-eroded grey (GM) and white (WM) matter VOIs as well as a mid-phantom cold spot (VOI_cold_) and VOIs from the Hammers atlas. In addition, left-right hemisphere differences and voxel-by-voxel differences compared to a reference image were assessed.

**Results:**

Systematic differences were observed for reconstructions with and without point-spread-function modelling (PSF_ON_ and PSF_OFF_, respectively). Normalising to image-derived activity, upper and lower limits ensuring image comparability were as follows: for PSF_ON_, RC_GMe_ = [0.97–1.01] and GMWMr_e_ = [3.51–3.91] for eroded VOI and RC_GM_ = [0.78–0.83] and GMWMr = [1.77–2.06] for non-eroded VOI, and for PSF_OFF_, RC_GMe_ = [0.92–0.99] and GMWMr_e_ = [3.14–3.68] for eroded VOI and RC_GM_ = [0.75–0.81] and GMWMr = [1.72–1.95] for non-eroded VOI.

**Conclusions:**

To achieve inter-scanner comparability, we propose selecting reconstruction settings based on RC_GMe_ and GMWMr_e_ as specified in “Results”. These proposed standards should be tested prospectively to validate and/or refine the harmonisation criteria.

**Supplementary Information:**

The online version contains supplementary material available at 10.1007/s00259-021-05201-w.

## Introduction

In clinical brain PET studies, images often are compared longitudinally or with a reference database. Rigorous quality control and assurance are required in order to prevent that variability and differences between PET systems with regard to image quality, can affect research conclusions or patient diagnostics. This is especially the case when the effects studied are small (e.g. annual change in amyloid signal in Alzheimer’s disease [1]), so that data from multiple centres need to be combined to form the large datasets needed to obtain statistically significant conclusions. The growing need for multi-centre collaborations and (raw) data sharing in clinical brain research [2, 3] further highlights the need for image comparability between PET systems across multiple clinical centres.

It is clear that, in addition to standardised procedures for data acquisition, harmonisation of image quality and quantification measures is required. With the current pace of technological advancements in scanner design and characteristics, performance between currently installed clinical PET systems varies considerably. Moreover, since imaging system vendors use proprietary software, selecting apparently equivalent acquisition and reconstruction settings will not necessarily yield equivalent results (if equivalent settings are available at all).

So far, multiple initiatives aiming to harmonise acquisition protocols and image quality procedures, specifically for brain PET imaging, have been published [4–8]. In addition to standard imaging quality assurance tests, proposed brain PET quality control and accreditation schemes include more stringent minimum requirements for image uniformity, noise, spatial resolution and image contrast [9]. Optimising reconstruction settings for individual PET systems has also been found to increase multi-centre comparability of PET images [4]. In addition, good results have been achieved by applying spatial smoothing to existing PET images to mitigate inter-scanner differences [10–12]. However, this method is not suitable for all studies, because small differences caused by pathology could also be reduced or even removed by this filtering step.

An alternative approach is to employ predefined harmonisation criteria similar to the EANM Research Ltd. (EARL) image quality standards developed for clinical oncology [13, 14], i.e. to select reconstruction settings for each individual scanner based on predetermined lower and upper quantitative performance limits as assessed by phantom imaging. This ensures that data from as many PET systems as possible can be combined, while maintaining image quality comparability.

Adherence to EARL standards does not necessarily ensure comparability of brain PET images. The NEMA NU 2 Body phantom used in these standards consists of multiple spheres with relatively high activity concentrations compared with the large uniform background compartment. While this geometry is suitable for simulating tumour uptake, tracer distribution in the brain generally is more uniform across larger compartments, i.e. grey matter (GM) and white matter (WM). This inherently leads to differences in characteristics important for accurate quantification (e.g. scatter correction, attenuation correction, partial volume effects). Therefore, in this study, the Hoffman 3D Brain phantom [15] was used to derive harmonisation standards. This commonly used and widely available standard phantom simulates brain uptake of flow or metabolism tracers (e.g. [^18^F]FDG) with a grey matter to white matter ratio (GMWMr) of 4.

The aim of this study was to establish harmonisation of image quality and quantification for brain PET imaging in a multi-centre setting by defining limits for quantitative performance criteria derived from Hoffman 3D Brain phantom images. Recognising that comparability between scanners is more important than achieving the theoretically correct quantitative value on each individual scanner, upper limits were defined in addition to lower limits. The maximum RC that the various clinical PET systems can achieve varies greatly. Therefore, if no upper limit were specified (or if the upper limit were set to the theoretical RC value), the differences in contrast recovery that would be permitted by the harmonisation criteria would be larger, which would lead to decreased comparability. Note, however, that we aimed to find the most accurate harmonising performance criteria, thus excluding systems that produced only reconstructed images of poor image quality so that these would not define the accreditation limits.

## Methods

A Hoffman 3D Brain phantom was scanned at seven different sites on a total of twelve different EARL-accredited PET/CT systems. Both analogue and SiPM-based “digital” systems of all three main PET/CT system vendors were included. Vendor and system characteristics are summarised in Table 1.

**Table 1:** PET/CT systems included in this study

Vendor	Model name	ID in graphs	SiPM-based “digital” systems?	Time-of-flight reconstruction available?	PSF reconstruction available?
GE	Discovery 690	1	N	Y	Y
	Discovery 710	2	N	Y	Y
	Discovery MI	3	Y	Y	Y
	Discovery MI	4	Y	Y	Y
Philips	Gemini	5	N	Y	N
	Ingenuity	6	N	Y	Y
	Vereos	7	Y	Y	Y
	Vereos	8	Y	Y	Y
Siemens	Biograph 40	9	N	Y	Y
	Biograph 64	10	N	Y	Y
	Horizon (trueV)	11	N	Y	Y
	Biograph 128 Vision	12	Y	Y	Y

### Phantom

The Hoffman 3D Brain phantom is an anthropomorphic phantom containing a 1140 mL compartment, representing the entire brain, to be filled with a radioactive solution. To simulate a grey matter to white matter ratio (GMWMr) of 4, the fillable volume is a factor of 4 smaller in areas representing WM than in areas representing GM. This is accomplished through placement of plastic layers within WM regions that are thin enough to be indiscernible on PET images (due to partial volume effects), leading to lower apparent activity concentrations. Given this design, in this paper, recovery coefficient (RC) is defined as the activity concentration measured by PET divided by the activity concentration of the *stock* solution (rather than the actual activity concentration in the GM and WM compartments). Theoretically, the recovery coefficients derived from the phantom PET image should therefore yield RC_GM_ = 1 and RC_WM_ = 0.25.

### Data acquisition

The phantom was filled from a 1500 mL stock solution containing ~ 50 MBq [^18^F]FDG (i.e. ~ 33 kBq/mL, similar to clinically observed GM activity concentrations for [^18^F]FDG brain PET) at the start time of each scan. A PET scan of at least 30 min (a duration chosen to minimise the influence of counting statistics on the results) was then acquired and reconstructed using various protocols and settings available on each system (Online Resource 1). Settings were chosen within a clinically relevant range. Where available, settings included the system’s proprietary clinical brain imaging protocol.

### VOI and mask definition

From the acquired dataset, one PET scan (matrix size 256 × 256 × 111 and voxel size 1.17 × 1.17 × 2.00 mm^3^) was selected to serve as template for defining PET-based volumes of interest (VOI). The accompanying CT scan was combined with two other CT scans from other systems, rigidly coregistered (using linear interpolation) to the first using Elastix software [16], to construct a maximum intensity CT image. The purpose of this step was to eliminate air bubbles from the image. The maximum intensity CT image was then used as template for defining CT-based VOI (matrix size 512 × 512 × 111 and voxel size 0.97 × 0.97 × 2.00 mm^3^).

#### CT-based VOI

First, spatial smoothing was applied to the CT template to smooth interfaces between compartments. Then the following VOI were defined: (a) a VOI representing the solid plastic areas within the phantom, which was automatically delineated with a HU > 95 threshold on the CT image; (b) a VOI representing WM which was automatically delineated with thresholds 50 < HU < 95; (c) a VOI representing GM which was automatically delineated with thresholds 0 < HU < 50]; (d) a left-right hemisphere binary mask which was defined manually using MATLAB [17]; and (e) a VOI representing the centrum semiovale region which was defined manually using ITK-snap [18]; for all automatically delineated VOI, small delineation errors were corrected manually using ITK-snap. All VOI and masks were resliced to PET template matrix size using Vinci [19]. In addition, a binary GM&WM mask was constructed to be used as the template for coregistration of the PET images. To prevent partial volume effects from influencing RC values, additional VOI were defined by eroding the GM and WM VOI using a sphere with 4-voxel radius (MATLAB function “imerode”). In the remainder, eroded VOI will be marked with subscript “e”.

#### PET-based VOI

(a) Using ITK-snap, two masks were defined manually onto the PET template: one generously encompassing the entire fillable compartment of the phantom (brain region + phantom edge) and one encompassing only the brain region; (b) in order to coregister the Hammers atlas [20], representing multiple brain regions, to PET template geometry, the binary GM&WM mask was scaled to resemble a T1 MR image. This facsimile MR image, together with the template PET, was then input into a PVE-lab pipeline [21] in order to generate the coregistered Hammers atlas. The resulting VOI were then segmented into GM and WM VOI using the GM&WM mask; (c) because the Hoffman phantom was designed to simulate [^18^F]FDG, it does not simulate all characteristics that could be relevant for brain PET imaging. For example, the phantom does not simulate a brain region of relatively low uptake (such as pons for flumazenil studies). Therefore, we have manually added a spherical VOI (2.59 mL) to the VOI atlas within a mid-phantom solid plastic area. This zero-uptake region in between regions of higher uptake is then used to simulate a mid-brain low uptake region (VOI_cold_).

### Image analysis

For each reconstructed PET image, the first three and last three axial slices were excluded to avoid field of view (FOV) edge effects. Subsequently, each scan was rigidly coregistered to the binary GM&WM map using Elastix and normalised to stock solution activity concentration to obtain RC images. Stock solution activity concentration was derived using two methods: (1) based on net phantom activity as measured by the locally available dose calibrator and (2) based on image-derived activity concentration, where whole-phantom activity was calculated from the image-derived activity in the phantom brain region, divided by the specified fillable volume of the phantom (i.e., 1140 mL) and multiplied by a correction factor. The latter estimates the fraction of brain region activity compared with the activity of the entire fillable compartment of the phantom (i.e. 0.92 ± 0.01, as derived from scans with sufficient axial FOV range to enable accurate quantification for the entire phantom compartment); for clarity, in the remainder of this paper, all measures derived from using method 1 will be indicated by superscript “dc”.

For each RC image, averaged RC values were derived from all VOIs. Eroded and non-eroded RC_GM_ and GMWMr results from all images were then evaluated to define upper and lower limits, such that at least one reconstructed image from each PET system produced values within these two sets of limits.

In addition, for each system, the reconstructed image that produced eroded and non-eroded RC_GM_ and GMWMr most closely approximating the mid-values between the limits was selected. In cases where different reconstructions were identified based on eroded and non-eroded RC_GM_ and GMWMr, the reconstruction with minimal total relative difference to the mid-values was selected. From the resulting set of images, an average and an SD image was constructed, to be used as reference for voxel-by-voxel comparison.

For all voxels within the GM&WM mask, differences between each RC image and the reference average image were compared to the reference SD image. The percentage of voxels with (absolute) differences >2SD was then calculated.

To enable fast and consistent evaluation of system compliancy, a software tool was developed for automated analysis of Hoffman 3D Brain phantom images, as outlined above, which includes all image masks, templates, reference images and harmonisation criteria. A description of the tool is given in Online Resource 2.

## Results

**Fig. 1:**
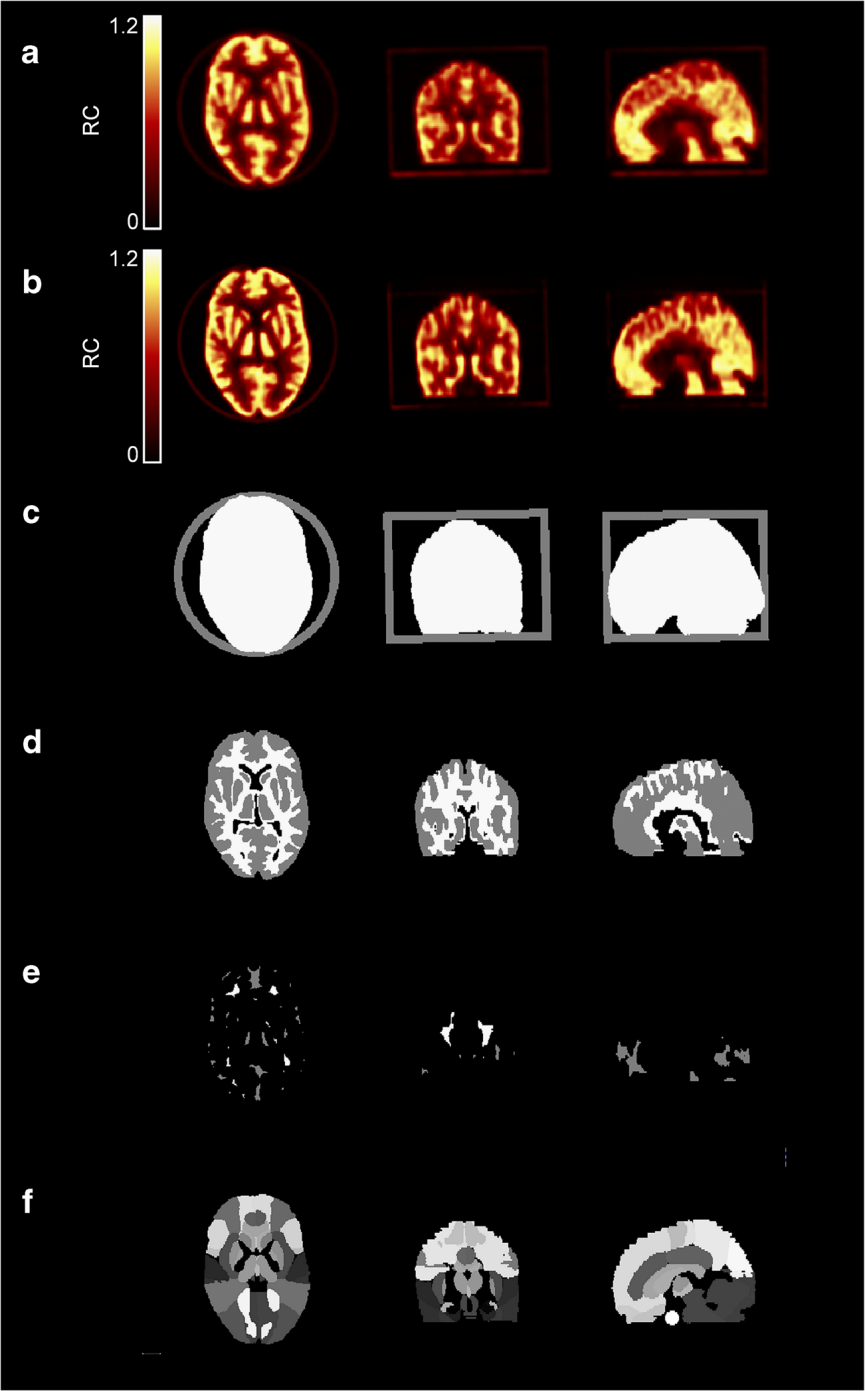
Typical RC-images for a PSF_OFF_ (**a**) and PSF_ON_ (**b**) reconstruction of a Hoffman 3D Brain phantom PET scan, along with the normalisation mask (**c**), the grey matter and white matter VOI (**d**), the eroded grey matter and white matter VOI (**e**) and the coregistered Hammers template with manually added VOI within an area not containing radioactive solution (**f**)

A total of 64 PET images were analysed. Figure 1 shows examples reconstructed with and without point-spread-function modelling (PSF_ON_ and PSF_OFF_, respectively), along with the image masks used for analysis.

**Fig. 2:**
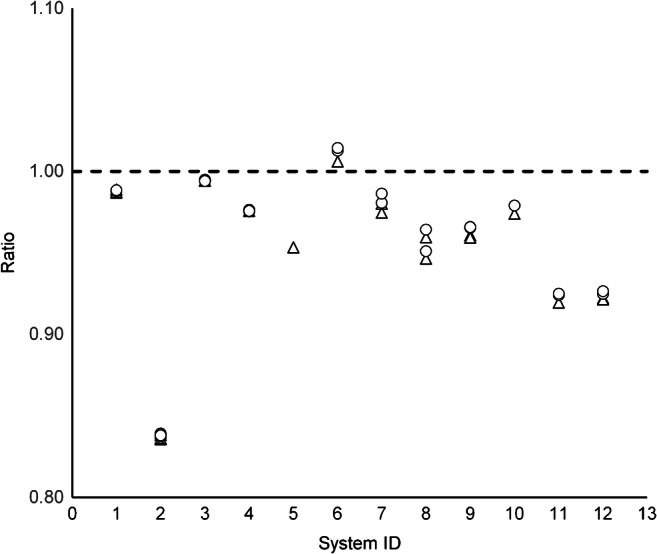
Ratio of image-derived versus dose calibrator-derived activity concentrations. Dose calibrator-derived values for system ID 2 were corrected for a suspected daylight savings error (original ratio ≈ 0.6)

The differences between image-derived and dose calibrator-derived activity concentrations are visualised in Fig. 2. For one PET system (system ID 2), unrealistically large differences were found that were inconsistent with the rest of the dataset (including those of the same scanner model). Correction for suspected daylight savings error could only partially resolve this issue. Image-normalised RC values were also relatively high compared to the full dataset. Visual inspection revealed all reconstructed images from this particular system to be of poor image quality. Therefore this system was excluded from the dataset that was used for developing quantitative harmonisation criteria and subsequently only used at a later stage to test whether those criteria succeeded in excluding all reconstructions from this system.

For the remaining reconstructions, dose calibrator-derived activity at PET start time was 38.05 ± 11.17 MBq, and ratios with image-derived activity (36.02 ± 7.65 MBq) were within a 10% range. Although this is consistent with the EARL criteria (that allow for a ± 10% cross-calibration error between dose calibrator and PET measurement), it is a wide range for brain imaging. Moreover, variation in dose calibrator-normalised RC (RC^dc^) was higher than for image-normalised RC: SD = 2.68% and 1.61%, respectively, for PSF_ON_ reconstructions and 2.86% and 2.19%, respectively, for PSF_OFF_ reconstructions, while for each system, variation between image-derived activity concentrations from the various reconstructions was small (SD < 1%). Therefore (and to prevent cross-calibration errors from affecting results), image-based normalised data were used for the remainder of this paper.

**Fig. 3:**
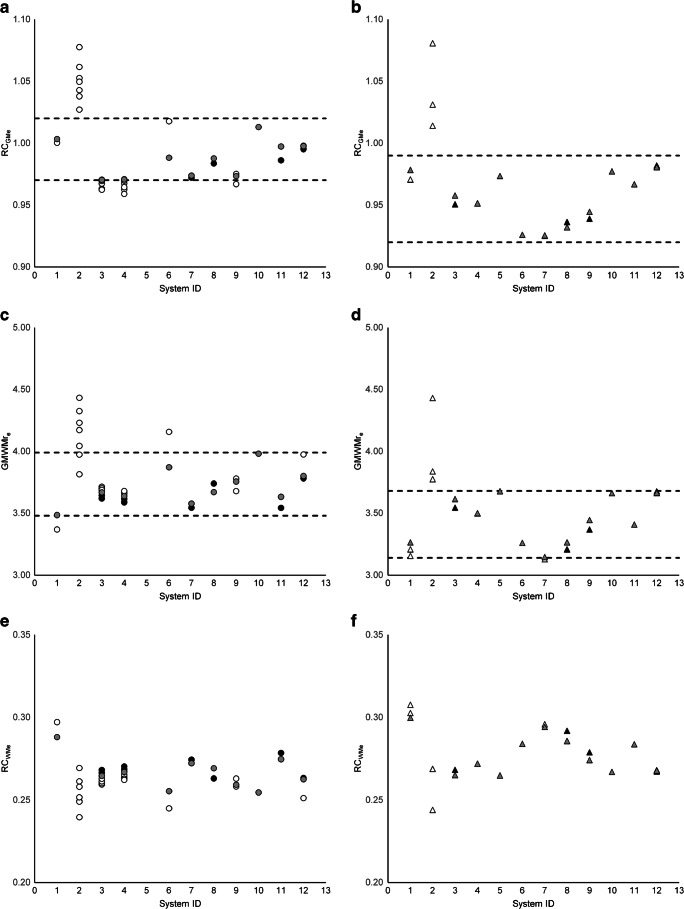
Eroded VOI RC_GMe_ (**a**, **b**) and GMWMr_e_ (**c**, **d**) and RC_WMe_ (**e**, **f**) for PSF_ON_ reconstructions (left column; circles) and PSF_OFF_ reconstructions (right column; triangles). Dashed lines represent the bandwidth, i.e. the proposed harmonisation criteria, such that for every system, at least one reconstruction shows results within the limits (excluding system ID 2). White, reconstructions not complying with both criteria; black, reconstructions complying with both criteria; grey, reconstructions selected for constructing the reference image

**Fig. 4:**
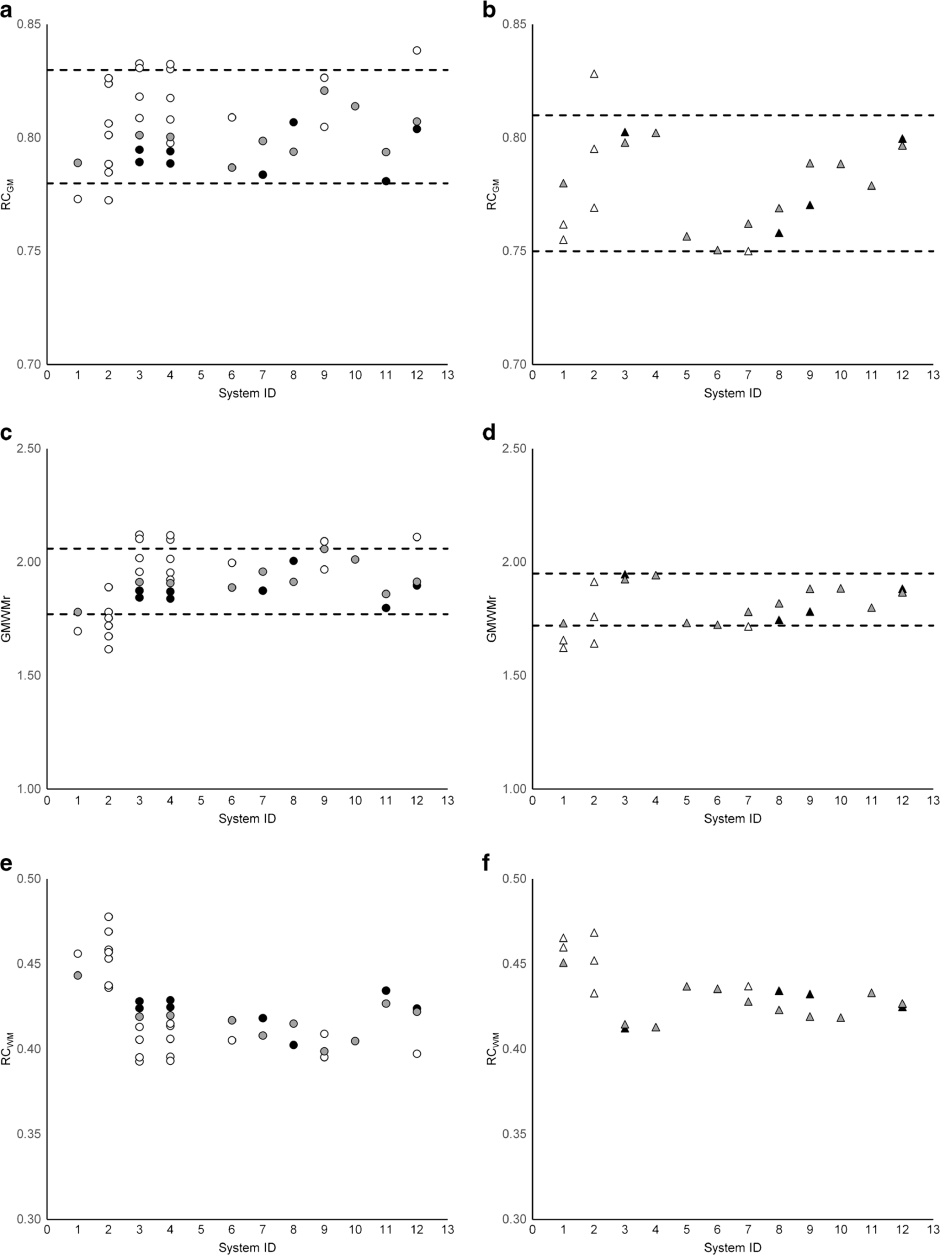
Non-eroded VOI RC_GM_ (**a**, **b**) and GMWMr (**c**, **d**) and RC_WM_ (**e**, **f**) for PSF_ON_ reconstructions (left column; circles) and PSF_OFF_ reconstructions (right column; triangles). Dashed lines represent the bandwidth, i.e. the proposed harmonisation criteria, such that for every system, at least one reconstruction shows results within the limits (excluding system ID 2). White, reconstructions not complying with both criteria; black, reconstructions complying with both criteria; grey, reconstructions selected for constructing the reference image

Systematic differences were observed for PSF_ON_ compared with PSF_OFF_ reconstructions (RCGM_e_= 0.98 ± 0.02 and GMWMr_e_= 3.70 ± 0.15 compared to RCGM_e_= 0.96 ± 0.02 and GMWMr_e_= 3.41 ± 0.21, where GE’s Q. Clear reconstruction was categorised as PSF_ON_; see Figs. 3 and 4). Therefore, in the remainder of this paper, results will be shown for PSF_ON_ and PSF_OFF_ reconstructions separately. No marked differences were observed in results for analogue versus digital PET/CT systems (RCGM_e_ = 0.98 ± 0.02 and GMWMre = 3.57 ± 0.27 compared to RCGM_e_ = 0.97 ± 0.02 and GMWMr_e_ = 3.61 ± 4.97).

Figures 3 and 4 show RC_GM_ and GMWMr for non-eroded and eroded GM and WM VOI, respectively, along with the limits for selecting reconstructions of comparable quality, chosen such that at least one reconstruction per system could adhere to both criteria (except for system ID 2, as mentioned above): for PSF_ON_, RC_GMe_ = [0.97–1.01] and GMWMr_e_ = [3.51–3.91] for eroded VOI and RC_GM_ = [0.78–0.83] and GMWMr = [1.77–2.06] for non-eroded VOI; and for PSF_OFF_, RC_GMe_ = [0.92–0.99] and GMWMr_e_ = [3.14–3.68] for eroded VOI and RC_GM_ = [0.75–0.81] and GMWMr = [1.72–1.95] for non-eroded VOI. In the figures, the actual reconstructions selected for creating the reference average and SD images for voxel-by-voxel analysis are marked in grey.

**Fig. 5:**
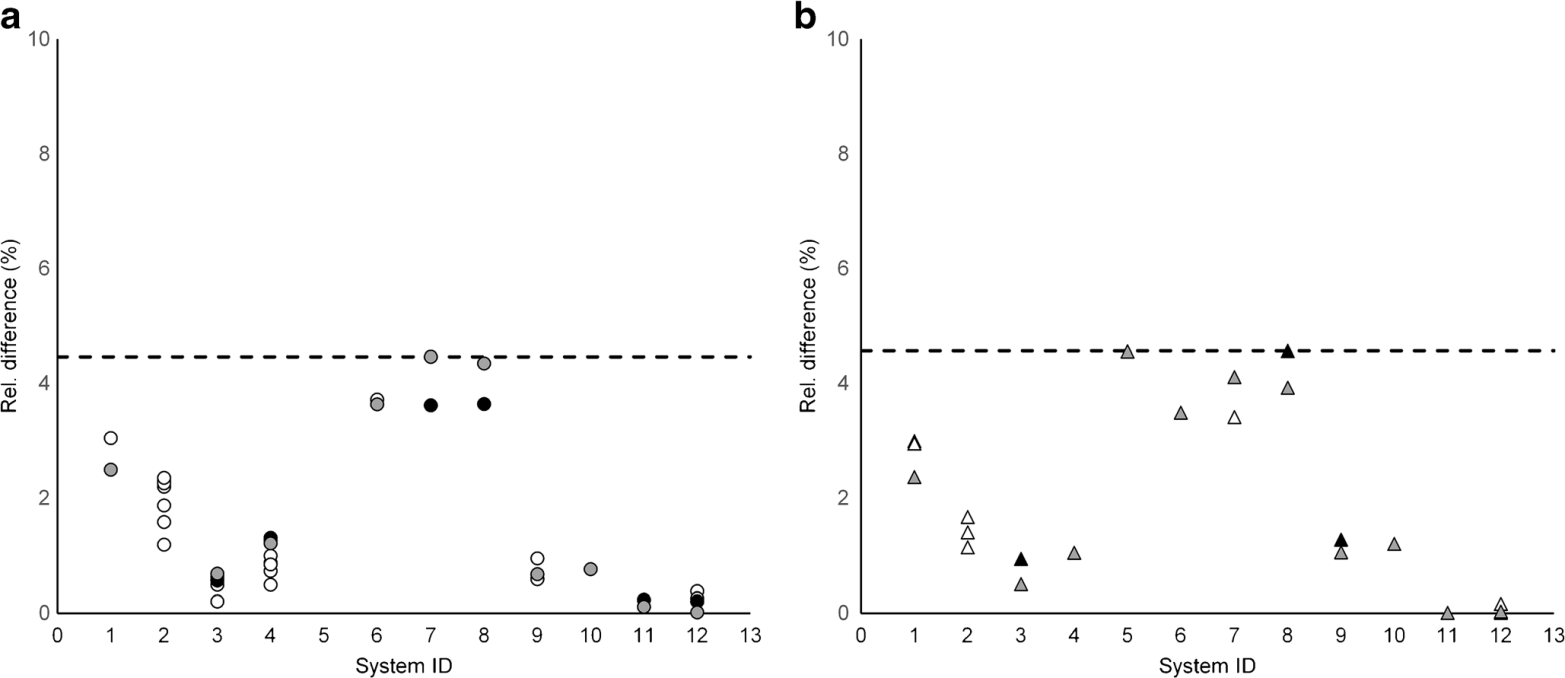
Absolute relative difference between RC_GM_ measured from left and right hemisphere for PSF_ON_ (**a**) and PSF_OFF_ (**b**) reconstructions. White, reconstructions not complying with both criteria; black, reconstructions complying with both criteria; grey, reconstructions selected for constructing the reference image. Dashed lines indicate range for reconstructions compliant to proposed criteria (Fig. 4). Note that for system ID 5, no PSF_ON_ reconstruction was available

**Fig. 6:**
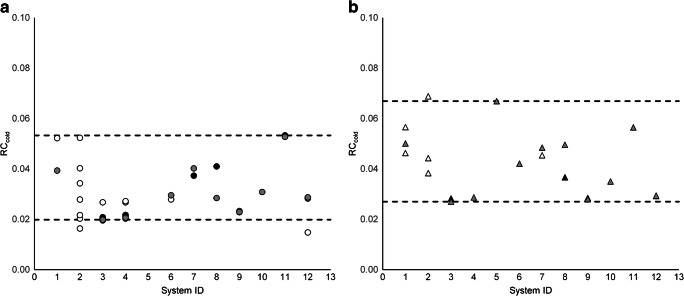
Cold spot RC for PSF_ON_ (**a**) and PSF_OFF_ (**b**) reconstructions. White, reconstructions not complying with both criteria; black, reconstructions complying with both criteria; grey, reconstructions selected for constructing the reference image. Dashed lines indicate range for reconstructions compliant to proposed criteria (Fig. 4)

**Fig. 7:**
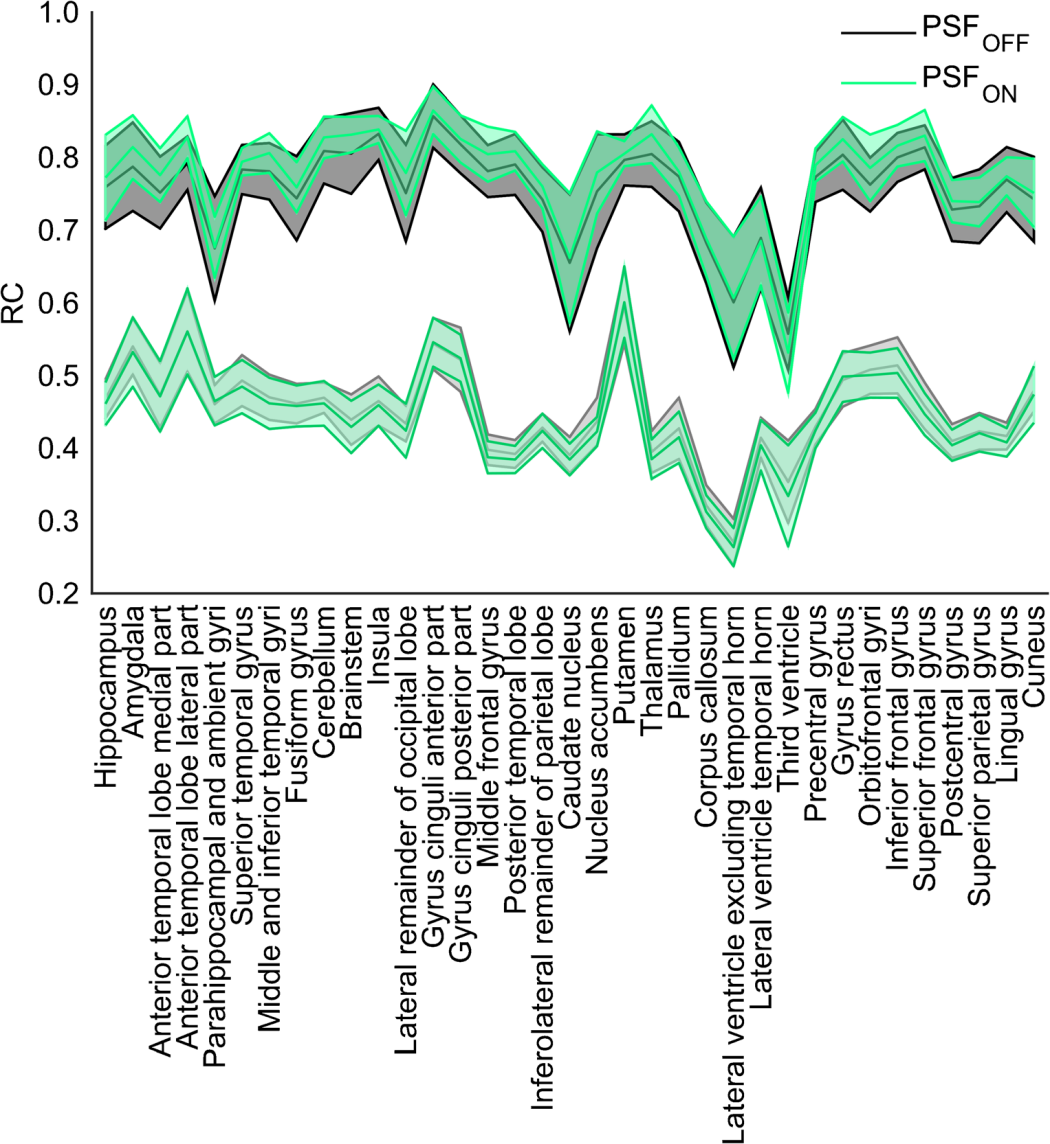
Hammers template mean RC_GM_ (top) and RC_WM_ (bottom) with confidence intervals (± 2SD) for PSF_ON_ and PSF_OFF_ reconstructions compliant with proposed criteria (Figs. 3 and 4). To reduce the number of VOI on the *x*-axis, RC values from left and right Hammers VOI were averaged for each structure

For reconstructions compliant with the proposed criteria (summarised in Tables 2 and 3), absolute relative differences between RC_GM_ derived from the left hemisphere and those derived from the right hemisphere were < 4.7% (range: 0.01–4.57%), as shown in Fig. 5. Systems from one vendor stood out with absolute relative differences of 3.95% ± 0.43% for PSF_ON_ and 4.03% ± 0.44% for PSF_OFF_, compared with 0.79% ± 0.67% and 1.19% ± 1.32%, respectively, for the other vendors. Figure 6 shows that RC from VOI_cold_ was in the range 0.02–0.05 and 0.03–0.07 for compliant PSF_ON_ and PSF_OFF_ reconstructions, respectively. In addition, RC_GM_ and RC_WM_ were derived for VOI from the Hammers template. Results are shown in Fig. 7, to be used as reference for future phantom analysis.

**Table 2:** Reconstruction settings for PSF_OFF_ reconstructions compliant with proposed harmonisation criteria

						Reconstruction method							
Vendor	System ID	Scanner model	Matrix size	Voxel size (mm^3^)	Scan duration (min)	Algorithm	PSF	TOF	Number of iterations	Number of subsets	*β*	Post-reconstruction filter (FWHM in mm)	Proprietary name
GE	1	Discovery 690	**128 × 128 × 47**	**2.73 × 2.73 × 3.27**	**30**	**OSEM**	**–**	**Y**	**4**	**16**		**–**	
	3	Discovery MI1	**256 × 256 × 71**	**0.98 × 0.98 × 2.79**	**40**	**OSEM**	**–**	**Y**	**3**	**34**		**3.0**†	**VUE Point FX**
			256 × 256 × 71	0.98 × 0.98 × 2.79	40	OSEM	–	Y	3	34		**–**	VUE Point FX
	4	Discovery MI2	**256 × 256 × 71**	**0.98 × 0.98 × 2.79**	**45**	**OSEM**	**–**	**Y**	**3**	**34**		**–**	**VUE Point FX**
Philips	5	Gemini	**128 × 128 × 90**	**2.00 × 2.00 × 2.00**	**30**	**RAMLA**	**–**	**Y**	**3**	**21**		**–**	**LOR-RAMLA**
	6	Ingenuity	**128 × 128 × 90**	**2.00 × 2.00 × 2.00**	**30**	**OSEM**	**–**	**Y**	**3**	**21**		**–**	**BLOB-OS-TF**
	7	Vereos	**256 × 256 × 164**	**1.00 × 1.00 × 1.00**	**30**	**OSEM**	**–**	**Y**	**3**	**15**		**4.1**	
	8	Vereos	**256 × 256 × 164**	**1.00 × 1.00 × 1.00**	**30**	**OSEM**	**–**	**Y**	**3**	**15**		1.0	
			128 × 128 × 82	2.00 × 2.00 × 2.00	30	OSEM	–	Y	3	15		2.0	
Siemens	9	Biograph 40	**400 × 400 × 148**	**2.04 × 2.04 × 3.00**	**30**	**OSEM**	**–**	**Y**	**5**	**21**		**–**	
			256 × 256 × 148	3.18 × 3.18 × 3.00	30	OSEM	–	Y	5	21		**–**	
	10	Biograph 64	**400 × 400 × 111**	**2.04 × 2.04 × 2.00**	**30**	**OSEM**	**–**	**Y**	**3**	**24**		**–**	
	11	Horizon (TrueV)	**360 × 360 × 111**	**2.06 × 2.06 × 2.00**	**30**	**OSEM**	**–**	**Y**	**4**	**10**		**–**	
	12	Biograph 128 Vision	512 × 512 × 175	0.71 × 0.71 × 3.00	30	OSEM	–	Y	4	5		**–**	
			**512 × 512 × 175**	**1.42 × 1.42 × 3.00**	**30**	**OSEM**	**–**	**Y**	**4**	**5**		**–**	

Bold font: reconstructions selected to create the reference image

*OSEM* ordered subset expectation maximization; *RAMLA* Row Action Maximum Likelihood Algorithm; † Transaxial filter, no axial filter

**Table 3:** Reconstruction settings for PSF_ON_ reconstructions compliant with proposed harmonisation criteria. NA: not available; OSEM: ordered subset expectation maximization; BSREM: block sequential regularized expectation maximization; * Transaxial filter with standard axial filter; † Transaxial filter, no axial filter

						Reconstruction method							
Vendor	System ID	Scanner model	Matrix size	Voxel size (mm^3^)	Scan duration (min)	Algorithm	PSF	TOF	Number of iterations	Number of subsets	*β*	Post-reconstruction filter (FWHM in mm)	Proprietary name
GE	1	Discovery 690	**128 × 128 × 47**	**2.34 × 2.34 × 3.27**	**30**	**OSEM**	**Y**	**Y**	**5**	**18**		3.0*	**VUE Point FX SharpIR**
	3	Discovery MI1	**256 × 256 × 71**	**0.98 × 0.98 × 2.79**	**40**	**BSREM**	**Y**	**Y**	**25**	**NA**	**200**	**–**	**Q.Clear**
			256 × 256 × 71	0.98 × 0.98 × 2.79	40	BSREM	Y	Y	25	NA	250	**–**	Q.Clear
			256 × 256 × 71	0.98 × 0.98 × 2.79	40	BSREM	Y	Y	25	NA	300	**–**	Q.Clear
	4	Discovery MI2	**256 × 256 × 71**	**0.98 × 0.98 × 2.79**	**45**	**BSREM**	**Y**	**Y**	**25**	**NA**	**200**	**–**	**Q.Clear**
			256 × 256 × 71	0.98 × 0.98 × 2.79	45	BSREM	Y	Y	25	NA	250	**–**	Q.Clear
			256 × 256 × 71	0.98 × 0.98 × 2.79	45	BSREM	Y	Y	25	NA	300	**–**	Q.Clear
Philips	6	Ingenuity	**128 × 128 × 90**	**2.00 × 2.00 × 2.00**	**30**	**OSEM**	**Y**	**Y**	**3**	**21**		**–**	**BLOB-OS-TF**
	7	Vereos	128 × 128 × 82	2.00 × 2.00 × 2.00	30	OSEM	Y	Y	3	15		4.1	
			**256 × 256 × 164**	**1.00 × 1.00 × 1.00**	**30**	**OSEM**	**Y**	**Y**	**3**	**15**		**4.1**	
	8	Vereos	256 × 256 × 164	1.00 × 1.00 × 1.00	30	OSEM	Y	Y	3	15		1.0	
			**128 × 128 × 82**	**2.00 × 2.00 × 2.00**	**30**	**OSEM**	**Y**	**Y**	**3**	**15**		2.0	
Siemens	9	Biograph 40	**512 × 512 × 148**	**1.59 × 1.59 × 3.00**	**30**	**OSEM**	**Y**	**Y**	**5**	**21**		**–**	**TrueX**
	10	Biograph 64	**400 × 400 × 111**	**2.04 × 2.04 × 2.00**	**30**	**OSEM**	**Y**	**Y**	**3**	**21**		**–**	**TrueX**
	11	Horizon (TrueV)	256 × 256 × 111	2.89 × 2.89 × 2.00	30	OSEM	Y	Y	4	10		**–**	TrueX
			**360 × 360 × 111**	**2.06 × 2.06 × 2.00**	**30**	**OSEM**	**Y**	**Y**	**4**	**16**		**–**	**TrueX**
	12	Biograph 128 Vision	**512 × 512 × 175**	**0.71 × 0.71 × 3.00**	**30**	**OSEM**	**Y**	**Y**	**4**	**5**		**–**	**TrueX**
			512 × 512 × 175	1.42 × 1.42 × 3.00	30	OSEM	Y	Y	4	5		**–**	TrueX

Bold font: reconstructions selected to create the reference image

*NA* not available; *OSEM* ordered subset expectation maximization; *BSREM* block sequential regularized expectation maximization; * Transaxial filter with standard axial filter; † Transaxial filter, no axial filter

**Fig. 8:**
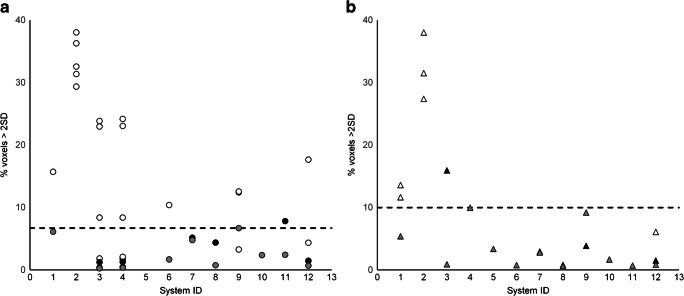
Voxelwise analysis results for PSF_ON_ (**a**) and PSF_OFF_ (**b**) reconstructions. White, reconstructions not complying with both criteria; black, reconstructions complying with both criteria; grey, reconstructions selected for constructing the reference image. Dashed line indicates proposed threshold

Results of the voxel-by-voxel analysis are shown in Fig. 8. For most reconstructions adhering to the criteria for RC_GM_ and GMWMr, the percentage of voxels with differences >2SD compared with the reference image was < 6.7% for PSF_ON_ and < 10.0% for PSF_OFF_. Exceptions were two PSF_OFF_ reconstructions from system ID1 that exhibited relatively large left-right hemisphere ratios and two PSF_ON_ reconstructions from system ID9.

## Discussion

This study focused on developing criteria for selecting PET reconstruction settings that can achieve quantitative performance harmonisation of brain PET studies across PET centres, so that data from different centres can be combined in order to generate the large datasets needed for detecting potentially small drug-induced changes. Similar to the EARL criteria in oncology, it is proposed to use lower and upper limits for RC_GM_ and GMWMr, as derived from imaging a Hoffman 3D Brain phantom, ensuring that for each system at least one reconstructed image complies with these criteria.

The present results indicate that PSF_ON_ and PSF_OFF_ reconstructions cannot be pooled. Interestingly, for PSF_ON_ reconstructions, RC_GMe_ > 1 were found, which theoretically should not be possible. This is not unexpected as PSF reconstructions are known to show Gibbs artefacts that can lead to local overestimation of activity concentrations [22]. For this reason, the application of PSF reconstruction for brain PET studies, that often require accurate quantification, is debatable. Regardless of the outcome of this debate, we have shown that harmonisation across PET/CT systems in terms of contrast recovery is possible for PSF reconstructions.

Errors in the image-derived stock activity concentration could also have affected RC values. As shown in Fig. 2, image-derived phantom activity concentrations were systematically lower than dose calibrator-derived phantom activity concentrations. This could be caused by voxels within the image mask not registering all relevant counts or by the slightly different estimate of the total phantom volume (i.e. 1140 mL). However, as the main objective of harmonisation criteria is to ensure image comparability, a systematic underestimation of total phantom activity is not problematic as long as the analysis is performed consistently. A remedy could be to normalise to the image-derived activity concentration within a GM VOI, assuming that RC_GM_ = 1 for this region, instead of to whole-phantom activity concentration. However, the potential presence of Gibbs artefacts or other non-uniformity issues within this VOI would subsequently change RC values for all other VOIs relative to the proposed harmonisation criteria and therefore change conclusions regarding the optimal reconstruction settings for that system. Therefore, a large region was chosen, capturing all activity in the phantom for deriving the stock activity concentration. In addition, the criteria for RC_GM_ were combined with that for GMWMr, a measure that is not affected by the stock activity concentration estimate.

Another solution would be to simply use the dose calibrator-derived normalisation. In that case, however, RC could be affected by (variable) errors in the dose calibrator to PET cross-calibration. Although these errors would be limited to ± 10% systems that comply with EARL criteria, this margin is too large for effects to be studied in the brain. In contrast, proposed RC_GM_ margins in the present study are ± 2.5% for PSF_ON_ and ± 3.7% for PSF_OFF_. Furthermore, brain PET analyses often use activity ratios relative to reference brain regions rather than dose calibrator-derived measures, in which case PET system calibration errors are not relevant.

In the present study, differences were observed in left-right hemisphere ratio across vendors (Fig. 5). Several systems (ID 5-8), all from the same vendor, exhibited relatively high left-right differences in RC_GM_, which indicates the presence of a gradient across the axial FOV. Phantom positioning was not consistently different for these systems. Therefore, this effect is most likely caused by vendor-specific reconstruction software. We hypothesise that it could be due to either a misalignment between PET and CT data, leading to errors in the attenuation correction map, or a problem with the TOF time alignment. Therefore, in case a gradient is observed, reviewing PET-CT spatial alignment and/or time alignment is recommended. Please note, however, that for scanners included in this manuscript, all the vendor recommended calibrations and normalisations were performed, and we were not able to resolve the issue nor gain understanding on the nature of this non-uniformity from our experiments nor from the vendor. The apparent gradient did not lead to high percentages in the voxel-by-voxel analysis (Fig. 8) though, which indicates that the magnitude of the gradient was small compared with other inter-scanner differences. In addition, no notable differences in variability for RC of the Hammers VOIs were observed between vendors. Nevertheless, when assessing clinical differences between left and right hemispheric regions, the observed ~ 4% difference between left and right hemisphere RC_GM_ should be taken into account.

Given that VOI_cold_ was defined within a zero-uptake region, theoretically RC_cold_ (as defined in “Methods”) should be equal to 0. From Fig. 6, it is clear that all scanners approximate RC_cold_ = 0. However, it is important to realise that RC from VOI_cold_ varied markedly between scanners: up to a factor of 2.7 for PSF_ON_ and 2.5 for PSF_OFF_ reconstructions (compared with a factor of 1.04 and 1.06, respectively, for RC_GMe_). We hypothesise that this is due to the different methods of scatter correction employed by the various scanners and possibly the OSEM non-negativity constraint. Of course, scanner performance could be optimised to yield better comparability of low-uptake recovery. However, this would likely affect comparability of recovery for other regions. This emphasises that quantification of very low uptake brain regions may not be feasible across scanners. Moreover, if such a very low uptake region were to be used as reference region, inter-scanner differences between calculated activity ratios could be substantial.

It is encouraging to see (as evident from Fig. 7) that for the reconstructions adhering to the proposed harmonisation criteria, the RC from the Hammers atlas VOI are similar across PET/CT systems. This indicates that by harmonising RC for the larger GM and WM VOI, the smaller sub-regions are also harmonised. Thereby, disease-specific brain patterns should be reproducible across PET/CT systems.

In theory, the criteria proposed for RC_GMe_ and GMWMr_e_ could be met accidentally, because any existing differences may be obscured by averaging over a large number of voxels within the VOI used (12,541 voxels, 34 mL, for eroded GM and 18,415 voxels, 50 mL, for eroded WM). Therefore, voxel-by-voxel analysis was included to capture potential regional quantitative biases. For this analysis, a reference image was generated by combining the reconstructions (one per system) that most closely approximated the mid-values between the upper and lower limits for RC_GMe_ and GMWMr_e_, i.e. those images with maximum comparability. To assess deviation from the reference, the number of voxels with RC values that were more than 2SD different from the reference image was measured. By using a threshold based on observed SD, voxels with reference image values of low precision were automatically excluded, reducing the influence of edge effects and small coregistration errors. The result from the current dataset seems to indicate that if over 10% (7% for PSF_ON_) of brain region voxels differ from the reference image by > 2SD, comparability should be considered questionable. It should be noted that the reference images were based on a limited dataset, not including all scanner models. As more data are collected, reference images could be adjusted, and more definitive thresholds can be derived.

As Joshi et al. pointed out, comparability increases when images are smoothed to the same (low) resolution [10]. While manipulating images in this way may be suitable for many applications, the increase in comparability comes at the cost of reduced spatial resolution and reduced accuracy. In some cases, particularly in those where expected effects are small, filtering may even remove the effect under study. Of course, by introducing upper limits to GMWMr_e_ and RC_GMe_, the harmonisation criteria proposed in the present study also lead to reduced resolution and accuracy for some scans, albeit less severe, because the upper limit leads to exclusion of some reconstructions that would have yielded higher recoveries, i.e. closer to the theoretical values of RC_GM_ = 1 and RCWM = 0.25. Inclusion of an upper limit was a deliberate choice made in order to keep the range between limits as small as possible, while ensuring all systems could qualify (excluding system ID 2). The aim of this study was to harmonise contrast recovery across PET/CT systems rather than to select those PET/CT systems that achieve the highest contrast recoveries. Note that proposed limits for RC_GMe_ are close to 1.0.

For studies requiring higher resolution and accuracy, more stringent harmonisation criteria may be needed. In some cases, the requirements may only become apparent retrospectively, which would require re-reconstruction of the data. Habert et al. showed that much can be gained in comparability by optimising settings for each scanner individually [4]. Similar to the approach in oncology, two reconstructions could be performed, one in line with the proposed harmonisation standards and a second one using the locally preferred or optimised settings [13].

Selecting reconstruction settings based on the proposed harmonisation criteria does not correct for technical issues of individual scanners. Therefore, in addition to those criteria, systems should comply with regular QC as well as EARL standards. Joshi et al. proposed to further improve comparability by retrospectively applying corrections to PET images tailored to each individual system. This could, for example, reduce the left-right hemisphere differences observed for some scanners. However, as evident from Fig. 8, for the data acquired in this study, it would not improve comparability across the dataset. For each case, the variability that a correction could introduce should be weighed against the magnitude of the error caused by the technical issue it corrects.

In addition, the analysis presented in this paper included only PET/CT systems and no PET/MR systems even though, in recent years, several PET/MR systems have been installed in clinics. Reason for this is that the technique involved to obtain the attenuation correction maps is completely different. While for PET/CT the attenuation map is based on a transmission image (from the CT), i.e. a direct measurement of attenuation, for PET/MR, the attenuation map needs to be derived from the MR image, which is particularly cumbersome for phantoms. However, it is worthwhile to explore the feasibility of including PET/MR in future (phantom) studies.

Another limitation of the present study is that the Hoffman 3D Brain phantom is intended to simulate the distribution of flow or metabolism tracers, such as [^18^F]FDG, in the brain. This means that, while the present results may be readily translatable to other tracers with a similar distribution pattern and GMWMr, the applicability for tracers with different distributions needs to be established. The Hoffman 3D Brain phantom was chosen as it is widely and commercially available, and its geometry is more appropriate for brain imaging than the commonly used NEMA NU 2 Body phantom.

In summary, in the present study, harmonisation criteria for brain PET studies were developed. To this end, scans of the Hoffman 3D Brain phantom acquired on 12 clinical PET systems were analysed, and RC and GMWMr results were compared for several VOIs. Lower and upper limits for both RC_GMe_ and GMWMr_e_ were selected such that each PET system included in the dataset could produce at least one reconstructed image that fulfilled these criteria. It should be noted that the proposed criteria need to be prospectively validated and/or further refined.

## Conclusions

In clinical brain imaging (research), comparability in image quality and quantification is of utmost importance. In this study, we developed criteria for selecting reconstruction settings to ensure optimal comparability of brain PET images across various PET systems. A combination of upper and lower limits to RC_GMe_ and GMWMr_e_, as derived from PET data acquired from the Hoffman 3D Brain phantom, was found to identify brain PET images of comparable quantitative performance. To enable quick and consistent evaluation of PET system compliancy, a software tool was developed for automated analysis. In summary, we have developed a prototype procedure with prototype criteria for harmonising PET system performances for brain studies. The procedure and criteria will be tested prospectively in the near future.

## Supplementary information

ESM 1 (PDF 875 kb)

ESM 2 (PDF 409 kb)

## Data Availability

Data presented in the figures and presented specifications are available upon request.
