# Supplementary material for: Harmonisation of PET/CT contrast recovery performance for brain studies
Source: Eur J Nucl Med Mol Imaging. 2021 Jan 31;48(9):2856–70. doi: 10.1007/s00259-021-05201-w (PMC8263427; doi:10.1007/s00259-021-05201-w)
Supplement: Supplementary file 1 — (PDF 875 kb) [file 259_2021_5201_MOESM1_ESM.pdf]

**Online resource material with: "Harmonisation of PET/CT contrast recovery performance for brain studies", European Journal of Nuclear Medicine and Molecular Imaging, 2021**
*Authors: E. E. Verwer[1], S. S. V. Golla[1], A. Kaalep[2,3], M. Lubberink[4], F. H. P. van Velden[5], V. Bettinardi[6], M. Yaqub[1], T. Sera[7,3], S. Rijnsdorp[8], A. A. Lammertsma[1], R. Boellaard[1]. [1] Amsterdam University Medical Centers, The Netherlands. [2] North Estonia Medical Centre Foundation, Estonia. [3] EANM Research Limited (EARL), Austria. [4] Uppsala University, Sweden. [5] Leiden University Medical Center, The Netherlands. [6] IRCCS Scientific Institute San Raffaele Hospital, Italy. [7] University of Szeged, Hungary. [8] Catharina Hospital Eindhoven, The Netherlands. Corresponding author: e.verwer@amsterdamumc.nl*

Table showing the reconstruction settings for each reconstructed PET image included in this study. Dark (light) shading indicates $PSF_{ON}$ ($PSF_{OFF}$) reconstructions complying with all harmonisation criteria (see Figs. 3 and 4 in the manuscript). Bold font: reconstructions selected for constructing the reference images for voxel-by-voxel analysis. na: not available, OSEM: ordered subset expectation maximization, BSREM: block sequential regularized expectation maximization, RAMLA: Row Action Maximum Likelihood Algorithm. * Transaxial filter with standard axial filter. † Transaxial filter, no axial filter

| | | | | | | Reconstruction method | | | | | | | |
|---|---|---|---|---|---|---|---|---|---|---|---|---|---|
| Vendor | System ID | Scanner model | Matrix Size | Voxel Size ($mm^3$) | Scan Duration (min) | Algorithm | PSF | TOF | Number of iterations | Number of subsets | β | Post-reconstruction filter (FWHM in mm) | Proprietary name |
| GE | 1 | Discovery 690 | **128 x 128 x 47** | **2.73 x 2.73 x 3.27** | **30** | **OSEM** | **-** | **y** | **4** | **16** | | **-** | |
| | | | 128 x 128 x 47 | 2.34 x 2.34 x 3.27 | 30 | OSEM | - | y | 3 | 18 | | 3.0* | VUE Point FX |
| | | | 128 x 128 x 47 | 2.34 x 2.34 x 3.27 | 30 | OSEM | - | y | 5 | 18 | | 3.0* | VUE Point FX |
| | | | 128 x 128 x 47 | 2.34 x 2.34 x 3.27 | 30 | OSEM | y | y | 3 | 18 | | 3.0* | VUE Point FX SharpIR |
| | | | **128 x 128 x 47** | **2.34 x 2.34 x 3.27** | **30** | **OSEM** | **y** | **y** | **5** | **18** | | **3.0*** | **VUE Point FX SharpIR** |
| | 2 | Discovery 710 | 128 x 128 x 47 | 2.34 x 2.34 x 3.27 | 30 | OSEM | y | y | 3 | 18 | | - | VUE Point FX SharpIR |
| | | | 128 x 128 x 47 | 2.34 x 2.34 x 3.27 | 30 | OSEM | - | y | 3 | 18 | | - | VUE Point FX |
| | | | 128 x 128 x 47 | 2.34 x 2.34 x 3.27 | 30 | BSREM | y | y | 25 | na | 600 | - | Q.Clear |
| | | | 128 x 128 x 47 | 2.34 x 2.34 x 3.27 | 30 | BSREM | y | y | 25 | na | 200 | - | Q.Clear |
| | | | 128 x 128 x 47 | 2.34 x 2.34 x 3.27 | 30 | BSREM | y | y | 25 | na | 350 | - | Q.Clear |
| | | | 128 x 128 x 47 | 2.34 x 2.34 x 3.27 | 30 | BSREM | y | y | 25 | na | 400 | - | Q.Clear |
| | | | 128 x 128 x 47 | 2.34 x 2.34 x 3.27 | 30 | BSREM | y | y | 25 | na | 800 | - | Q.Clear |
| | | | 128 x 128 x 47 | 2.34 x 2.34 x 3.27 | 30 | OSEM | - | y | 3 | 18 | | 4.9† | VUE Point FX |
| | | | 128 x 128 x 47 | 2.34 x 2.34 x 3.27 | 30 | OSEM | - | y | 3 | 18 | | - | VUE Point FX SharpIR |
| | 3 | Discovery MI1 | **256 x 256 x 71** | **0.98 x 0.98 x 2.79** | **40** | **OSEM** | **-** | **y** | **3** | **34** | | **3.0†** | **VUE Point FX** |
| | | | 256 x 256 x 71 | 0.98 x 0.98 x 2.79 | 40 | OSEM | y | y | 3 | 34 | | - | VUE Point FX SharpIR |
| | | | 256 x 256 x 71 | 0.98 x 0.98 x 2.79 | 40 | OSEM | - | y | 3 | 34 | | - | VUE Point FX |
| | | | 256 x 256 x 71 | 0.98 x 0.98 x 2.79 | 40 | BSREM | y | y | 25 | na | 50 | - | Q.Clear |
| | | | 256 x 256 x 71 | 0.98 x 0.98 x 2.79 | 40 | BSREM | y | y | 25 | na | 100 | - | Q.Clear |
| | | | 256 x 256 x 71 | 0.98 x 0.98 x 2.79 | 40 | BSREM | y | y | 25 | na | 150 | - | Q.Clear |
| | | | **256 x 256 x 71** | **0.98 x 0.98 x 2.79** | **40** | **BSREM** | **y** | **y** | **25** | **na** | **200** | - | Q.Clear |
| | | | 256 x 256 x 71 | 0.98 x 0.98 x 2.79 | 40 | BSREM | y | y | 25 | na | 250 | - | Q.Clear |
| | | | 256 x 256 x 71 | 0.98 x 0.98 x 2.79 | 40 | BSREM | y | y | 25 | na | 300 | - | Q.Clear |
| | 4 | Discovery MI2 | 256 x 256 x 71 | 0.98 x 0.98 x 2.79 | 45 | OSEM | y | y | 3 | 34 | | 3.0† | VUE Point FX SharpIR |
| | | | 256 x 256 x 71 | 0.98 x 0.98 x 2.79 | 45 | BSREM | y | y | 25 | na | 100 | - | Q.Clear |
| | | | 256 x 256 x 71 | 0.98 x 0.98 x 2.79 | 45 | BSREM | y | y | 25 | na | 150 | - | Q.Clear |
| | | | **256 x 256 x 71** | **0.98 x 0.98 x 2.79** | **45** | **BSREM** | **y** | **y** | **25** | **na** | **200** | - | Q.Clear |
| | | | 256 x 256 x 71 | 0.98 x 0.98 x 2.79 | 45 | BSREM | y | y | 25 | na | 250 | - | Q.Clear |
| | | | 256 x 256 x 71 | 0.98 x 0.98 x 2.79 | 45 | BSREM | y | y | 25 | na | 300 | - | Q.Clear |
| | | | 256 x 256 x 71 | 0.98 x 0.98 x 2.79 | 45 | BSREM | y | y | 25 | na | 50 | - | Q.Clear |
| | | | **256 x 256 x 71** | **0.98 x 0.98 x 2.79** | **45** | **OSEM** | **-** | **y** | **3** | **34** | | **-** | **VUE Point FX** |
| | | | 256 x 256 x 71 | 0.98 x 0.98 x 2.79 | 45 | OSEM | y | y | 3 | 34 | | - | VUE Point FX SharpIR |
| | | | 256 x 256 x 71 | 0.98 x 0.98 x 2.79 | 45 | OSEM | y | y | 3 | 34 | | 3.0† | VUE Point FX SharpIR |
| Philips | 5 | Gemini | **128 x 128 x 90** | **2.00 x 2.00 x 2.00** | **30** | **RAMLA** | **-** | **y** | **3** | **21** | | **-** | **LOR-RAMLA** |
| | 6 | Ingenuity | **128 x 128 x 90** | **2.00 x 2.00 x 2.00** | **30** | **OSEM** | **-** | **y** | **3** | **21** | | **-** | **BLOB-OS-TF** |
| | | | **128 x 128 x 90** | **2.00 x 2.00 x 2.00** | **30** | **OSEM** | **y** | **y** | **3** | **21** | | **-** | **BLOB-OS-TF** |
| | | | 128 x 128 x 90 | 2.00 x 2.00 x 2.00 | 30 | OSEM | y | y | 3 | 21 | | - | BLOB-OS-TF |
| | 7 | Vereos | 128 x 128 x 82 | 2.00 x 2.00 x 2.00 | 30 | OSEM | - | y | 3 | 15 | | 4.1 | |
| | | | 128 x 128 x 82 | 2.00 x 2.00 x 2.00 | 30 | OSEM | y | y | 3 | 15 | | 4.1 | |
| | | | **256 x 256 x 164** | **1.00 x 1.00 x 1.00** | **30** | **OSEM** | **-** | **y** | **3** | **15** | | **4.1** | |
| | | | **256 x 256 x 164** | **1.00 x 1.00 x 1.00** | **30** | **OSEM** | **y** | **y** | **3** | **15** | | **4.1** | |
| | 8 | Vereos | 256 x 256 x 164 | 1.00 x 1.00 x 1.00 | 30 | OSEM | y | y | 3 | 15 | | 1.0 | |
| | | | **128 x 128 x 82** | **2.00 x 2.00 x 2.00** | **30** | **OSEM** | **y** | **y** | **3** | **15** | | 2.0 | |
| | | | **256 x 256 x 164** | **1.00 x 1.00 x 1.00** | **30** | **OSEM** | **-** | **y** | **3** | **15** | | 1.0 | |
| | | | 128 x 128 x 82 | 2.00 x 2.00 x 2.00 | 30 | OSEM | - | y | 3 | 15 | | 2.0 | |
| Siemens | 9 | Biograph 40 | **400 x 400 x 148** | **2.04 x 2.04 x 3.00** | **30** | **OSEM** | **-** | **y** | **5** | **21** | | - | |
| | | | 256 x 256 x 148 | 3.18 x 3.18 x 3.00 | 30 | OSEM | - | y | 5 | 21 | | - | |
| | | | 400 x 400 x 148 | 1.02 x 1.02 x 3.00 | 30 | OSEM | y | y | 5 | 21 | | - | TrueX |
| | | | 400 x 400 x 148 | 2.04 x 2.04 x 3.00 | 30 | OSEM | y | y | 5 | 21 | | - | TrueX |
| | | | 256 x 256 x 148 | 3.18 x 3.18 x 3.00 | 30 | OSEM | y | y | 5 | 21 | | - | TrueX |
| | | | **512 x 512 x 148** | **1.59 x 1.59 x 3.00** | **30** | **OSEM** | **y** | **y** | **5** | **21** | | - | **TrueX** |
| | 10 | Biograph 64 | **400 x 400 x 111** | **2.04 x 2.04 x 2.00** | **30** | **OSEM** | **-** | **y** | **3** | **24** | | **-** | |
| | | | **400 x 400 x 111** | **2.04 x 2.04 x 2.00** | **30** | **OSEM** | **y** | **y** | **3** | **21** | | **-** | **TrueX** |
| | 11 | Horizon (TrueV) | 256 x 256 x 111 | 2.89 x 2.89 x 2.00 | 30 | OSEM | y | y | 4 | 10 | | - | TrueX |
| | | | **360 x 360 x 111** | **2.06 x 2.06 x 2.00** | **30** | **OSEM** | **-** | **y** | **4** | **10** | | **-** | |
| | | | **360 x 360 x 111** | **2.06 x 2.06 x 2.00** | **30** | **OSEM** | **y** | **y** | **4** | **16** | | **-** | **TrueX** |
| | 12 | Biograph 128 Vision | **512 x 512 x 175** | **0.71 x 0.71 x 3.00** | **30** | **OSEM** | **y** | **y** | **4** | **5** | | **-** | **TrueX** |
| | | | 512 x 512 x 175 | 0.71 x 0.71 x 3.00 | 30 | OSEM | - | y | 4 | 5 | | - | |
| | | | **512 x 512 x 175** | **1.42 x 1.42 x 3.00** | **30** | **OSEM** | **-** | **y** | **4** | **5** | | **-** | |
| | | | 440 x 440 x 175 | 1.65 x 1.65 x 3.00 | 30 | OSEM | - | y | 4 | 5 | | - | |
| | | | 512 x 512 x 175 | 1.42 x 1.42 x 3.00 | 30 | OSEM | y | y | 4 | 5 | | - | TrueX |
| | | | 440 x 440 x 175 | 1.65 x 1.65 x 3.00 | 30 | OSEM | y | y | 4 | 5 | | - | TrueX |
| | | | 440 x 440 x 159 | 1.65 x 1.65 x 1.65 | 30 | OSEM | y | y | 8 | 5 | | - | TrueX |
