# Supplementary material for: Harmonisation of PET/CT contrast recovery performance for brain studies
Source: Eur J Nucl Med Mol Imaging. 2021 Jan 31;48(9):2856–70. doi: 10.1007/s00259-021-05201-w (PMC8263427; doi:10.1007/s00259-021-05201-w)
Supplement: Supplementary file 2 — (PDF 409 kb) [file 259_2021_5201_MOESM2_ESM.pdf]

Online resource material with: "Harmonisation of PET/CT contrast recovery performance for brain studies", European Journal of Nuclear Medicine and Molecular Imaging, 2021
Authors: E. E. Verwer[1], S. S. V. Golla[1], A. Kaalep[2,3], M. Lubberink[4], F. H. P. van Velden[5], V. Bettinardi[6], M. Yaqub[1], T. Sera[7,3], S. Rijnsdorp[8], A. A. Lammertsma[1], R. Boellaard[1]. [1] Amsterdam University Medical Centers, The Netherlands. [2] North Estonia Medical Centre Foundation, Estonia. [3] EANM Research Limited (EARL), Austria. [4] Uppsala University, Sweden. [5] Leiden University Medical Center, The Netherlands. [6] IRCCS Scientific Institute San Raffaele Hospital, Italy. [7] University of Szeged, Hungary. [8] Catharina Hospital Eindhoven, The Netherlands. Corresponding author: e.verwer@amsterdamumc.nl

Description of the Hoffman Phantom Analysis Tool

The HoffmanPhantomAnalysisTool can be used to consistently analyse PET scans of the 3D Hoffman Brain phantom and test compliancy to the harmonisation criteria proposed in this paper.

Fig. A shows a screen capture of the tool's user interface. The tool accepts PET scans in ECAT or NifTi format. After loading the scan and filling out the necessary scan information, the tool will coregister the scan to a predefined template, normalize the image to image-derived total phantom activity concentration and derive RC as well as GMWMr for various VOI. It will also compare the scan to a predefined reference image and calculate the extent of deviation from this image.

The tool will then report the most important results and compare the values to the predefined harmonisation criteria. The tool will display "Pass" (green), "Fail" (red) or "Warning" (orange) depending on the result. If none of the results show "Fail", the scan is considered compliant to the harmonisation criteria. Full results are stored in .csv as well as .mat files. Several displaying options are available for QC purposes.

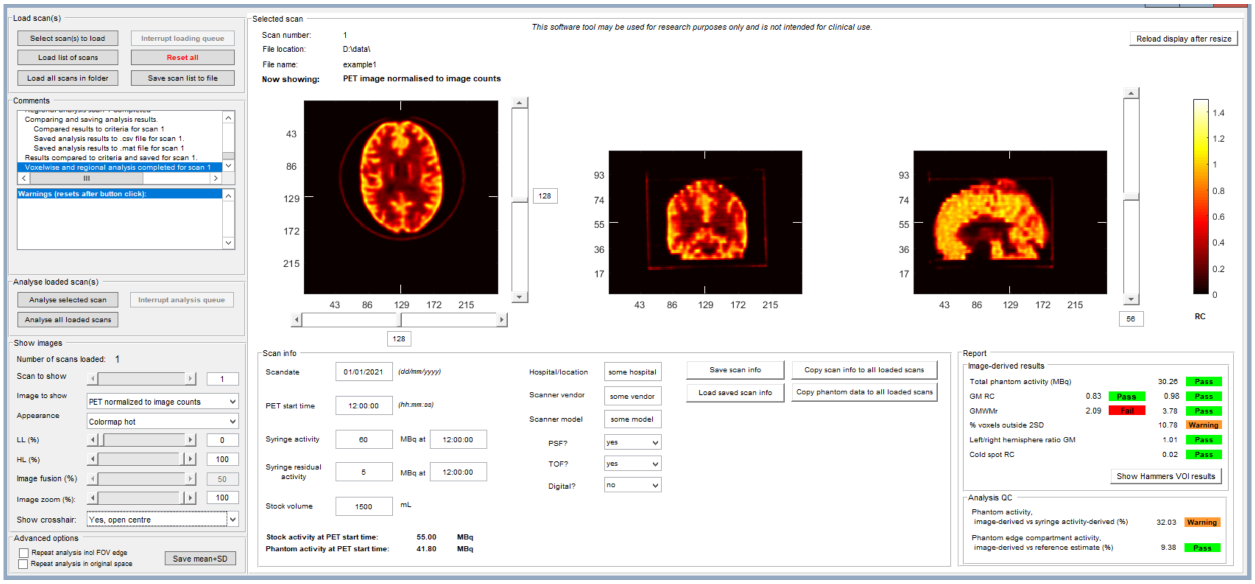

Fig A. Screencapture of the user interface for the HoffmanPhantomAnalysisTool showing an example of an analysed PET image (in this case not complying with harmonisation criteria).
